# Deep SVDD and Transfer Learning for COVID-19 Diagnosis Using CT Images

**DOI:** 10.1155/2023/6070970

**Published:** 2023-03-07

**Authors:** Akram A. Alhadad, Reham R. Mostafa, Hazem M. El-Bakry

**Affiliations:** ^1^Computer Science Department, Ibb University, Ibb, Yemen; ^2^Information Systems Department, Faculty of Computers and Information, Mansoura University, Mansoura 35511, Egypt

## Abstract

The novel coronavirus disease (COVID-19), which appeared in Wuhan, China, is spreading rapidly worldwide. Health systems in many countries have collapsed as a result of this pandemic, and hundreds of thousands of people have died due to acute respiratory distress syndrome caused by this virus. As a result, diagnosing COVID-19 in the early stages of infection is critical in the fight against the disease because it saves the patient's life and prevents the disease from spreading. In this study, we proposed a novel approach based on transfer learning and deep support vector data description (DSVDD) to distinguish among COVID-19, non-COVID-19 pneumonia, and intact CT images. Our approach consists of three models, each of which can classify one specific category as normal and the other as anomalous. To our knowledge, this is the first study to use the one-class DSVDD and transfer learning to diagnose lung disease. For the proposed approach, we used two scenarios: one with pretrained VGG16 and one with ResNet50. The proposed models were trained using data gathered with the assistance of an expert radiologist from three internet-accessible sources in end-to-end fusion using three split data ratios. Based on training with 70%, 50%, and 30% of the data, the proposed VGG16 models achieved (0.8281, 0.9170, and 0.9294) for the F1 score, while the proposed ResNet50 models achieved (0.9109, 0.9188, and 0.9333).

## 1. Introduction

COVID-19 is the common name of the disease caused by the novel coronavirus (2019-nCoV). The infected patients will mostly have mild to severe respiratory illnesses. Although most patients can recover without the need to take certain medications, some will get very ill and require medical treatment. People with chronic diseases such as cardiovascular disease, diabetes, chronic respiratory disease, or cancer, in addition to those over 65 years of age, are more likely to develop serious illnesses. People of different ages can become very ill or die due to the infection [1]. COVID-19 has impacted every aspect of our everyday lives since it spreads fast and causes acute respiratory distress syndrome [2]. Infected people in the initial stage have numerous typical symptoms, including headaches, fevers, coughs, and severe pain in most muscles of the body [3, 4]. Many patients perished as a result of severe pneumonia and breathing difficulties [5]. The COVID-19 virus not only has a physical impact but also causes mental illnesses such as anxiety and depression, which can also result in death [6, 7]. Therefore, many studies have been conducted to analyze the sentiments of people using Twitter to prevent further health deterioration [8].

The rapid spread of COVID-19 necessitates early detection of infected individuals to combat the disease and save patients' lives [9]. The polymerase chain reaction (PCR) is the gold-standard test to identify COVID-19; however, it may yield incorrect results in the initial stage of the illness and typically requires 5–6 hours [10].

Furthermore, the scarcity of PCR kits has resulted from the increase in infected people, creating a new problem. As a result, the timely diagnosis and treatment of patients will stop the spread of COVID-19. As a result, the use of CT received attention, and numerous studies were carried out to verify its efficacy as a substitute for identifying infected individuals [10]. These studies reveal that most patients' CT images have consolidation spots and ground-glass opacities (GGO), indicating that CT scans may be used to verify the presence of COVID-19 disease and monitor therapeutic progression [10, 11]. The coronavirus spreads quickly between people or when in contact with an area where an infected person resides. To avoid erroneous diagnoses, a team of radiologists must review the CT [12].

As a result of the rapid development of vision techniques, numerous vision models have been used to detect anomalies in medical images and identify tumors. Researchers have proposed several techniques for segmenting lesions in the skin, brain, and other tissues. These systems utilized a variety of medical images, including CT scans, ultrasounds, X-rays, and magnetic resonance imaging (MIR) [13, 14]. These techniques provide amazing results, sometimes superior to those of radiologists [15].

Several studies have used CT images and deep learning (DL) to identify COVID-19 and discover lung abnormalities [16, 17]. DL techniques can extract the appropriate features, which, in turn, increases the effectiveness of the models they use. This is why almost all of the proposed approaches use them to detect coronavirus in CT scans. On the other hand, DL techniques require large amounts of data to train deep models. Therefore, the lack of data makes it difficult to train a deep model to detect COVID-19 using CT scans [18].

Despite the encouraging results of these studies in identifying COVID-19, additional enhancements are necessary to address some limitations. The lack of balanced and sufficient data, intraclass variation, and interclass similarity represent the main obstacles to building a robust system. In addition, since Corona is a new virus, the researchers gathered CT images from several sources, so most of them are blurry, and there is also a high variation between them. As a result, if a model were developed to categorize CT scans as COVID-19 or healthy, all lung abnormalities would be labeled as COVID-19, which is not medically accurate.

To address the aforementioned challenges, a novel approach using deep SVDD and transfer learning is proposed to identify COVID-19. Two different pretrained models (VGG16 and ResNet50) are used separately with deep SVDD. Our approach is composed of three models. Each model can recognize a particular class. In general, and specifically for COVID-19 diagnostics, we believe that we are the first to use pretrained models with DSVDD. The following is a summary of the research's contributions:

- With the help of a radiology specialist, we constructed a new dataset including three classes (intact, COVID-19, pneumonia) of CT images from three available resources on the Internet.
- We proposed a new approach based on the one-class DSVDD consisting of three models. Each model is trained to find a closed hypersphere combining only one class of samples. As a result, when a new virus is discovered, we simply train a new model on the newly discovered virus data without affecting the other models.
- The basic deep network in the original DSVDD was replaced with pretrained VGG16 and ResNet50 networks to address the data shortage, and then we trained the models using each network separately.

The remainder of this work is structured as follows: Section 2 represents the related work. A short overview of all the concepts and techniques utilized in our research is provided in Preliminaries Section 3. In Section 4, we describe the dataset used in this research. In Section 5, the proposed approach is explained in detail.  Section 6 outlines the experiments and results in detail. We conclude this work in Section 7.

## 2. Related Work

Numerous studies have proposed DL techniques to identify COVID-19. These studies addressed the data deficiency by enhancing data, transferring learning, or designing a new neural network structure. A three-branched deep neural network (DNN) based on the ResNet50 structure was proposed by Wu et al. [19]. The network receives three different views of the CT images, one for each branch. The extracted features for each branch are fused and passed to fully connected layers to determine whether they are COVID-19 or not. Using ten pretrained models; Ardakani et al. [20] identified COVID-19 pneumonia and non-COVID pneumonia in the cropped lesion regions of CT images. A radiologist must crop the infected areas in the proposed framework. Amyar et al. [21] suggested a three-task DNN. This network starts with an encoder, which receives CT images and generates output that is used in three branches to classify, segment, and reconstruct the CT image. The U-Net structure is employed to segment and reconstruct the CT image. Gozes et al. [22] used the U-Net to clip the lung parenchyma. The region of interest was then classified as intact or COVID-19-infected using pretrained ResNet50. Wu et al. [23] designed a DNN to identify COVID-19 and determine the infection area using CT scans. The Res2Net is used as the base classification model after replacing the last dense layer with a two-channel layer representing the probability of COVID-19 and intact patients. The segmentation model is made up of encoders and decoders based on VGG16 and the U-Net style. Yousefzadeh et al. [24] used the pretrained EfficientNetB3 model to build a system to classify all slices of a CT file into normal, COVID-19, and non-COVID. This system used the features extracted from the trained model to train fully connected layers. Wang et al. [25] propose a two-stage framework. First, the lung lobes were extracted using 3D-UNET. In the second stage, two subnetworks are used to categorize the extracted lobes. While one subnetwork is trained to identify pneumonia, the other predicts the kind of pneumonia. Hu et al. [26] proposed a deep model to identify COVID-19 using CT scans. A trained 3D U-Net is used to segment the parenchyma of a normalized CT scan. This model is inspired by the VGG architecture, where all convolution blocks use a kernel of size 3 × 3. Because the COVID-19 lesion regions change depending on severity, multi-scale learning is utilized. The multiscale features from multiple levels are combined to represent the network's final output. Li [27] proposed a framework for distinguishing COVID-19, community-acquired pneumonia, and nonpneumonia. It is a deep 3D model that extracts 2D and 3D features based on ResNet50. This model receives sequences of CT slices to generate the feature maps. These feature maps are combined using a max pooling layer and then passed to a fully connected layer, which uses the SoftMax activation function to generate probability scores for each class. Using CT scans, Wang et al. [28] presented a method for diagnosing COVID-19 using CT scans. First, manually obtain the ROI (region of interest). The ROIs are sent to the preprocessing stage, where images are converted to grayscale and the smallest rectangle enclosing the parenchyma is extracted using thresholding and flood fill techniques. The method used the pretrained Inception model for the classification task. Using transfer learning and trained models GoogleNet, ResNet, and AlexNet, Zhou et al. [29] built the ensemble model (EDL_COVID). These models are initialized with the pretrained weights to act as feature extractors. This model used the relative majority vote algorithm and Softmax for the final outputs. Fan et al. [30] proposed a model with two branches: one that extracts local features using VGG19 and the other extracts global features using a transformer. The extracted local and global features are fused using a bidirectional fusion model that connects the two branches. The proposed model classifies the input images as intact, COVID-19, and non-COVID-19 pneumonia. We summarized the related work in Table 1.

## 3. Preliminaries

This section briefly describes the techniques used and illustrates some notations.

### 3.1. Support Vector Data Description (SVDD) [31]

SVDD is an OC-SVM-related technique that is used to solve one-class classification problems. In the one-class task, typically only positive class data are available, whereas the negative class data are either unavailable or insufficient to train a binary classifier. The idea behind SVDD is to separate positive data by a hypersphere characterized by a radius *ℛ* > 0 and a center *C* ∈ *F*. Most positive samples in the feature space *F* are grouped by this hypersphere. Let *X*⊆*ℝ*^*d*^ is the space of the inputs, *φ*_*κ*_(*x*) : *X*⟶*F*_*κ*_ is a transfer function with kernel Κ : *X* × *X*⟶[0, *∞*), *F*_*κ*_ is the space of the dot product.

Given positive training samples *D*  = {*x*_1_, *x*_2_,……………., *x*_*n*_}, *x*_*i*_ ∈ *X*, , *i* = 1,2,………..*n*, *n* refers to the sample count. Following is a formalization of the SVDD problem:

(1) $$\begin{array}{c} \begin{array}{c} \min\\ R,∁,\xi \end{array}\;R^{2}+\frac{1}{\upsilon n}\underset{i}{\sum }\xi_{i}\text{,}\\ s.t{\left\|\varphi_{\kappa }\left(x_{i}\right)-∁\right\|}_{F_{k}}^{2}\leq R^{2}+\xi_{i},\xi_{i}\geq 0,\forall i\text{.} \end{array}$$

To allow the soft margin, slake variable *ξ* is used, *υ* ∈ (0,1] controls the trade-off between penalties *ξ*_*i*_ and the volume of the sphere. When a point *x* meets the following criteria, it represents an outlier point:

(2) $$\begin{array}{c} {\left\|\varphi_{\kappa }\left(x\right)-∁\right\|}_{F_{k}}^{2}>R^{2}\text{.} \end{array}$$

### 3.2. Deep Learning (DL)

DL is a branch of machine learning that uses techniques to mimic the activity of the human brain neocortex. The neocortex is made up of layers of neurons that make up 80% of the wrinkly brain and is where most thinking occurs [32]. These layers are learned in a hierarchical order in which degrees of abstract representation are learned to interpret data patterns. Low and many levels of data abstraction represent high-level abstractions. The ability of deep learning to automatically learn how to extract features at various abstract levels is one of its most potent capabilities. This property allows us to develop systems that are able to locate complex transfer functions without the requirement for handcrafted features [32]. Building DNNs involves stacking layers vertically and learning from a big dataset. During the learning of a DNN, the weights and biases are updated via the backpropagation algorithm to minimize the prediction error. DNN layers learn feature extraction in a hierarchical manner, where the lower layer learns low-level features such as edges, while the higher layer learns high-level features such as eyes, noses, and faces [33].

### 3.3. Deep Support Vector Data Description (DSVDD)

**Figure 1:**
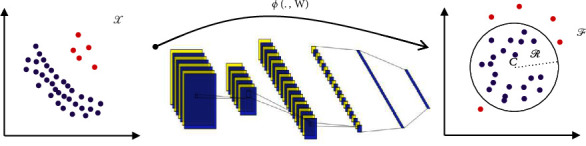
Through the training of DSVDD, the DCNN is trained and the *W* is updated to transfer the samples from *𝒳*⊆*ℝ*^*m*^ to *ℱ*⊆*ℝ*^*d*^ and then look for the minimum-volume hypersphere. This hypersphere is characterized by radius *ℛ* and center *C*. It is supposed to enclose representations of most training samples generated by the transformer *ϕ*(∙, *W*). The red points are anomalies because they are not inside the hypersphere.

Most researchers are drawn to DL because of its recent success in almost all areas of computer science. Ruff [31] proposed the DSVDD model to detect abnormal images. DSVDD is a hybrid of the deep convolutional neural network (DCNN) and SVDD. The DSVDD looks for the minimum-volume hypersphere that encloses the transferred data generated by the DCNN. The authors proposed two models: soft-margin and one-class models. The one-class model reduces the hypersphere's volume by minimizing the mean squared distance between the hypersphere's center and all training samples. It is intended for this hypersphere to include only normal images, as shown in Figure 1, so images outside of it are categorized as abnormal.

### 3.4. Transfer Learning

Big datasets are often required for DNN training, but building large datasets can be time-consuming and occasionally impossible because of a data shortage. Transfer learning (TL) is a strategy for transferring knowledge obtained by DNNs such as DCNNs to tackle another related challenge. Since COVID-19 is a new disease, the lack of adequate data is the biggest obstacle. Therefore, TL and DCNN are considered significant techniques for building intelligent systems that aid in diagnosing the disease. The TL process starts by initializing a pretrained DCNN model with weights obtained previously by training on a large dataset. These initial weights allow the DCNN model to extract features that can be adapted to perform a variety of tasks. Two strategies are used to adapt pretrained DCNN to a new task [34]. The first employs the pretrained model as a feature extractor. The model architecture is unchanged, except for the classification components, which are replaced by a different classification structure. In this strategy, only the classification parts are trained using the current task's dataset, whereas the feature extractor part is still the same [35]. The second strategy involves adapting the pretrained DCNN architecture to our current problem [34].

## 4. Dataset

**Figure 2:**
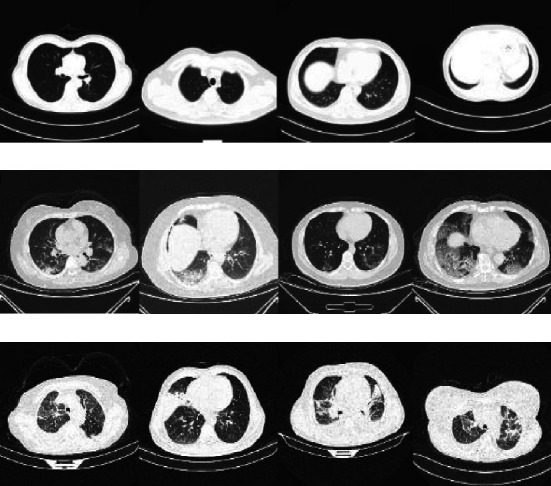
(a): Intact, (b) COVID-19, and (c) non-COVID19 pneumonia are samples from our dataset.

Building a deep-learning system to identify COVID-19 in the absence of sufficient data is a difficult task. As a result, most of the studies used clinical data to train their deep models. We constructed a CT dataset by gathering data from three resources on the Internet. This dataset contains images categorized into COVID-19, non-COVID-19 pneumonia, and intact. The CT scan files are made up of a series of slices. If the files belong to an infected patient, healthy slices must be removed, and nonparenchymal slices must be removed from all CT files [36]. As a result, an experienced radiologist reviewed these datasets to eliminate unwanted slices. The COVID-CT dataset [37] has two sets of slices. The first set contains 349 slices from COVID-19 patients, while the second set contains 397 slices from non-COVID-19 lung infections. To supplement the data, two additional datasets are used. COVID_DATASET [38] has three sets: COVID-19 (719 slices), intact (2495 slices), and pneumonia (1825 slices). Finally, the SARS-CoV-2 CT dataset [39] is split into two categories: 1252 COVID-19 CT slices and 1230 non-COVID-19 CT slices. We removed the non-COVID CT from the previous two datasets because they could contain a wide range of lung disorders, and we wanted to create a dataset with only three categories.  Figure 2 shows some examples from our dataset.

Furthermore, we also used COVID-19 [40], which consists of 20 COVID-19 CT scan files (NII.gz files) with ground truth files. The ground truth files are divided into three groups of masks: one for the entire parenchyma, including the lesion regions; another for the lesion areas only; and a third for the parenchyma. We used this dataset to train U-Net [41] for parenchyma extraction. Details about these datasets are shown in Table 2.

## 5. Proposed Approach

**Figure 3:**
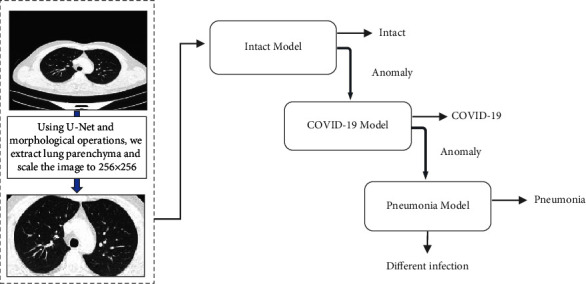
A trained U-Net extracts the parenchyma. Morphological operations are used to remove undesirable portions. The parenchyma is then classified using the intact model. If the parenchyma is abnormal, the COVID-19 model will be used to determine if the infection is COVID-19. If the parenchyma is impaired and does not contain COVID-19, the pneumonia model will classify it as pneumonia or another disease. Each model is built based on a one-vs-all approach, i.e., the intact model is trained on healthy samples, but during the testing and validation, we use samples from all classes to make sure about the ability of the trained model to distinguish between healthy samples and the others. In the same way, the COVID-19 and pneumonia models are trained, validated, and tested.

The proposed approach, including the preprocessing stage and deep classification models, is described in detail in this section.  Figure 3 depicts the proposed approach.

The diagnostic process in the proposed approach starts with the CT being classified as healthy or infected using the intact model; if the CT is infected, it is passed to the COVID-19 model for classification as COVID-19, or it is passed to the pneumonia model for classification as pneumonia or another type of infection. To develop our approach, we proposed two methods: one that used pretrained VGG16 with DSVDD and the other that used pretrained ResNet50 with DSVDD, where each method was built separately. For all models (intact, COVID-19, and pneumonia), the first method uses the VGG16 with DSVDD as the classifier, whereas the second uses ResNet50 with DSVDD. All stages and models of the proposed approach will be explained in detail in the following sections:

### 5.1. Preprocessing Stage

Preprocessing is a crucial step in the development of DL systems. The constructed dataset's slices came from a variety of sources. Therefore, the proposed method may not work well on most of these slices due to various edges close to the lung tissue. We trained the U-Net model to extract the parenchymal mask. Morphological operations are carried out on the mask to remove any possible small areas close to the lung parenchyma. To obtain the parenchyma, we utilized the extracted mask. Finally, we scaled all slices to 256 × 256 pixels, and the color was adjusted to fall within the range [0, 1].

### 5.2. Proposed Method

We proposed two methods for identifying COVID-19 based on DSVDD [31], The first method combines DSVDD with the pretrained ResNet50 [42], and the second with the pretrained visual geometry group network VGG16 [43]. We will go over the proposed methods in detail in this section:

#### 5.2.1. The Modified Pretrained VGG16

The original VGG16 is built with fixed-size 3 × 3 convolution filters and trained on millions of images with 1000 classes (ImageNet). It has a simple architecture but is very effective at the same time. The small 3 × 3 field filter can learn discriminative features of biomedical images at a fine-grained level and can gain information in a small area around the center [43, 44]. VGG16 uses thirteen convolutional layers to extract features and three dense layers for classification [43, 45]. The name beyond “VGG16” is that it has sixteen layers with learnable parameters [45]. VGG16 can extract a wide range of features due to the variety of classes it was trained on. As a result, it is applicable to a wide range of classification tasks.

**Figure 4:**
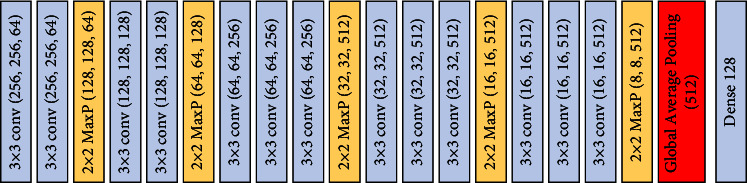
The modified pretrained VGG16.

In our classification task, we used the pretrained VGG16 after replacing the last three dense layers with a global average pooling layer and a dense layer with only 128 neurons, as illustrated in Figure 4.

#### 5.2.2. The Modified Pretrained ResNet50

**Figure 5:**
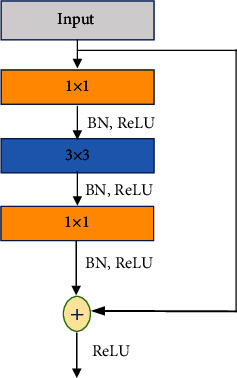
The residual block used in ResNet50.

The design of very deep networks causes a problem of gradient vanishing and degradation. This issue is resolved by residual learning using the ResNet developed by He et al. [42]. In the proposed method, we used the pretrained ResNet50 after replacing the last dense layers with a global average pooling layer and a dense layer with only 128 neurons. ResNet50 is made up of multiple stacked residual blocks, conventional convolution, and pooling layers. Each residual block combines three convolution layers of 1 × 1, 3 × 3, and 1 × 1 kernels, respectively, with a skip connection that adds the inputs of the first layer to the outputs of the last layer in the block, as depicted in Figure 5. Each convolution layer in the residual block is followed by batch normalization (BN) and the rectified linear activation function (ReLU), respectively [46].

#### 5.2.3. Deep Support Vector Data Description (DSVDD) with Pretrained Models

There are abnormal areas when comparing CT slices of COVID-19 patients with those of healthy individuals. We can consider this abnormal area an anomaly compared to healthy areas. The severity of the infection enlarges the anomalous regions [10].

In this study, DSVDD with pretrained models (VGG16 and ResNet50) is proposed to detect anomalous CT. The proposed DSVDD addresses data shortages while also improving COVID-19 diagnosis. We proposed two methods. DSVDD-VGG, which combines the pretrained VGG16 with DSVDD, and DSVDD-ResNet, which combines the pretrained ResNet50 with DSVDD. The pretrained models serve as feature extractors, while DSVDD seeks the minimum-volume hypersphere containing the extracted features of the normal slices.

Let Χ = {*𝓍*_1_, *𝓍*_2_,……………, *𝓍*_*n*_}, Χ⊆*ℝ*^*𝓂*^ be a set of the normal data used in the training process. *φ*(∙, *𝕎*) : Χ⟶ *𝔽*, *𝔽*⊆*ℝ*^*𝒹*^ are the pretrained models,*𝕎* = {*𝓌*^1^, *𝓌*^2^,……………, *𝓌*^*ℓ*^} represents the weights of all layer in the pretrained model, *𝓌*^*ℓ*^ are the weights of the layer *ℓ* ∈ {1,2,………., *L*}, *L* represents the number of layers. The proposed DSVDD aims to initialize VGG16 and ResNet50 with the trained weights and retrain them on our dataset to locate the minimum-volume hypersphere surrounding the samples that belong to the normal class. We formulate below the objectives of DSVDD-VGG and DSVDD-ResNet:

(3) $$\begin{array}{c} \begin{array}{c} \min\\ W \end{array}\;\frac{1}{n}\;\overset{n}{\underset{i=1}{\sum }}{\left\|\varphi \left(x_{i};W\right)-c\right\|}^{2}+\frac{t}{2}\overset{L}{\underset{l=1}{\sum }}{\left\|w^{l}\right\|}_{F}^{2}\text{.} \end{array}$$

The length between the extracted features *φ*(*𝓍*_*i*_; *𝕎*) and *𝒸* ∈ *𝔽* is penalized using the quadratic loss function. The second term is a network weight decay regularizer with a hyperparameter *t*>0. In order to reduce the hypersphere's volume, DSVDD minimizes the mean distance between the hypersphere's center and the extracted features. A given *𝓍* ∈ Χ is considered an anomalous slice if its length exceeds the last radius *R^∗^* from the hypersphere's center *𝒸* s.t

(4) $$\begin{array}{c} score\left(x\right)={\left\|\varphi \left(x;W^{*}\right)-c\right\|}^{2}\text{,} \end{array}$$

where *𝕎^∗^* is the final trained network's weights

(5) $$\begin{array}{c} \left\{\begin{array}{c} x\;is\;normal,score\left(x\right)\leq R^{*}\text{,}\\ x\;is\;anomoly,score\left(x\right)>R^{*}\text{.} \end{array}\right. \end{array}$$

### 5.3. Implementation Outline

Because our dataset has three categories, we trained three models of DSVDD-VGG and three models of DSVDD-ResNet independently. During each model's training, one category was labeled “normal” and the other “anomalous.” Due to restricted resources, we use a batch size of four images to train our models. We developed our approach using the Keras and TensorFlow libraries. We trained each model for 50 epochs using the NVIDIA Tesla K80 GPU. The Adam optimizer was used, with an initial learning rate of (10^−5^) and *β*1, *β*2 were set to 0.9 and 0.999, respectively.

In terms of training the U-Net model, we used 90% of the data for 50 epochs to train the standard architecture U-Net model. The remaining data was used to evaluate the model during training to select the best-trained model. We used the Adam optimizer with a batch size of 10 and an initial learning rate of (10^−4^). Augmentation with (rotation = 0.2, width and height shift = 0.05, shear = 0.05, zoom = 0.05) was used to increase the training data.

## 6. Experiments

### 6.1. Training and Testing Strategies

We trained one model of DSVDD-VGG and DSVDD-ResNet independently for each class in our dataset. We trained each model to classify one category as normal and the other as anomalous. To further investigate the efficacy of our proposed models, three different split ratios were used to train all models: 70%, 50%, and 30% of all categories were used for training, 10% of all categories were used for validation, and the remaining samples were used for testing.

The intact model considers the healthy CT slice as normal, while the pneumonia and COVID-19 slices are anomalous. Similarly, the pneumonia and COVID-19 models consider their slices as normal while the others are anomalous. We used only positive samples for training because we used a one-class classifier, but we used both positive and negative samples for validation and testing. Let's take a look at the intact model training process with 70% of the data as an example.

We trained the model with intact samples and validated it after every epoch with samples from healthy, COVID-19, and pneumonia slices. The trained model was tested on data from all classes that had not been included in training and validation. The checkpoint was saved when the current validation's evaluation metrics values exceeded the previous highest validation value.

### 6.2. Evaluation Metrics

Specificity, sensitivity, area under the receiver operator curve (AUC), and the F1 score were utilized to assess the proposed models. Specificity indicates how well the model distinguishes negative cases, whereas sensitivity indicates how well the model distinguishes positive cases. Because we were dealing with highly unbalanced data for binary classification tasks, the AUC metric and F1 score were utilized to assess each model's overall performance. The following are the formulas for the metrics used:

(6) $$\begin{array}{c} Specificity=\frac{TN}{\left(TN+FP\right)}\text{,}\\ Sensitivity=\frac{TP}{\left(TP+FN\right)}\text{,}\\ Precision=\frac{TP}{\left(TP+FP\right)}\text{,}\\ Recall=\frac{TP}{\left(TP+FN\right)}\text{,}\\ F1\;score=\frac{\left(2*Recall*Precision\right)}{\left(Recall+Precision\right)}\text{,} \end{array}$$

where FP = False Positive, FN = False Negative, TP = True Positive, and TN = True Negative.

### 6.3. Results

The testing and validation results for all data split ratios for the DSVDD-VGG and DSVDD-ResNet models are shown in the following:

#### 6.3.1. Intact Models

**Figure 6:**
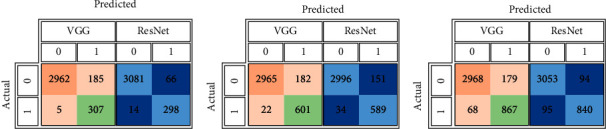
The intact DSVDD-VGG and DSVDD-ResNet models' confusion matrices correspond to the used partitions' percentages. (a) The intact models' confusion matrices based on the training using 70% of the healthy slices. (b) The intact models' confusion matrices based on the training using 50% of the healthy slices. (c) The intact models' confusion matrices based on the training using 30% of the healthy slices.

**Figure 7:**
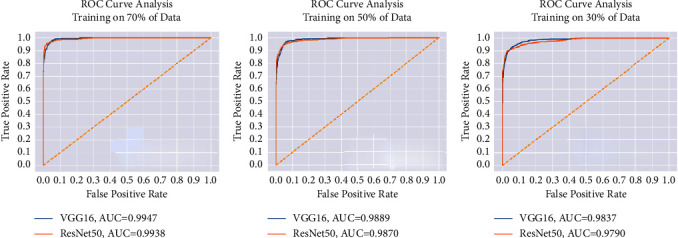
The intact models' ROC for each training scenario. (a) ROC when training using 70% of the intact slices. (b) ROC when training using 50% of the intact slices. (c) ROC when training using 30% of the intact slices.

For each data split ratio, Table 3 depicts the validating and testing evaluation metrics of the intact DSVDD-VGG and DSVDD-ResNet models. The results revealed that our models could accurately identify intact CT slices regardless of the number of training samples used. The intact model's confusion matrices for validation and testing are depicted in Figure 6.  Figure 7 depicts the receiving operating characteristic (ROC) curves for the intact models. The ROCs indicate that the models are stable and perform well.

**Figure 8:**
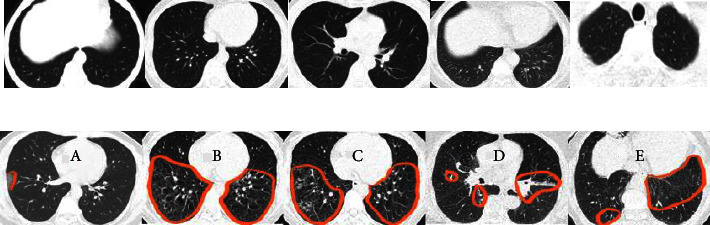
Examples of misclassified slices by the intact models. (a) Intact slices were categorized as anomalous. (b) Infected slices were categorized as intact slices. Marked areas represent: in (A) GGO (B, C) GGO and fibrotic bands (FB) (D) FB and (E) GGO.

According to the confusion matrices, DSVDD-VGG trained on 70% of the intact data, correctly classified all intact slices except five, and had the highest sensitivity metric value of any intact model. The specificity values indicate that the DSVDD-ResNet, which was trained on 70% of the intact data, produced better results regarding separating anomalous CT. In terms of overall evaluation, F1 score values show that DSVDD-ResNet has fewer false positives than DSVDD-VGG, but DSVDD-VGG has a higher AUC value, indicating that it is more sensitive to intact slices.  Figure 8 exhibits examples of CT slices that were misclassified using intact models.

#### 6.3.2. COVID-19 Models

**Figure 9:**
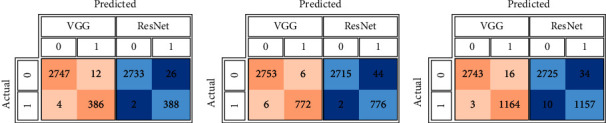
The COVID-19 DSVDD-VGG and DSVDD-ResNet models' confusion matrices corresponding to the used partitions' percentages. (a) The COVID-19 models' confusion matrices based on the training using 70% of the COVID-19 slices. (b) The COVID-19 models' confusion matrices based on the training using 50% of the COVID-19 slices. (c) The COVID-19 models' confusion matrices based on the training using 30% of the COVID-19 slices.

**Figure 10:**
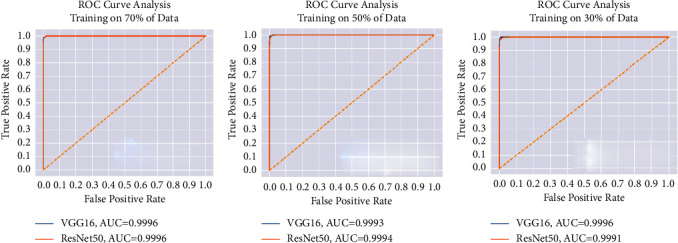
The COVID-19 models' ROC for each training scenario. (a) ROC when training using 70% the COVID-19 slices. (b) ROC when training using 50% of the COVID-19 slices. (c) ROC when training using 30% the COVID-19 slices.

Table 4 summarizes the values of the COVID-19 model metrics for validation and testing according to the different training ratios. The confusion matrices in Figure 9 indicate that the models efficiently discriminate COVID-19 slices from other categories, with specificity and sensitivity values close to 99%.  Figure 10 depicts the ROC curves of the COVID models.

**Figure 11:**
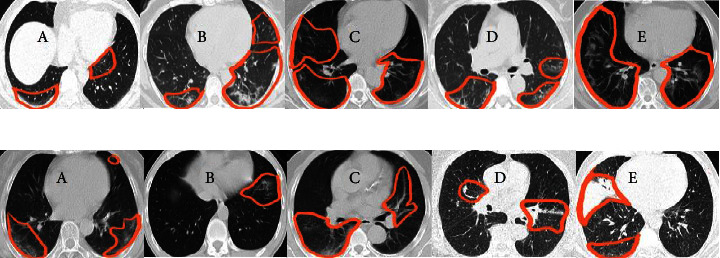
Examples of misclassified slices by the COVID-19 models. (a) COVID-19 CT slices were classified as non-COVID-19. Marked areas represent: in (A) GGO (B) GGO and consolidation (C–E) GGO. (b) Non-COVID slices were classified as COVID-19. Marked areas represent: in (A, B) GGO (C) GGO and vascular enlargement (D, E) FB.

Figure 11 exhibits examples of misclassified slices.  Figure 11(a) depicts samples of COVID slices that are classified as non-COVID. In Figure 11(b), non-COVID slices are classified as COVID-19 slices.

#### 6.3.3. Pneumonia Models

**Figure 12:**
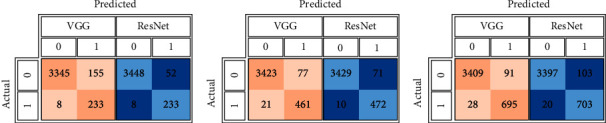
The pneumonia DSVDD-VGG and DSVDD-ResNet models' confusion matrices corresponding to the used partitions' percentages. (a) The pneumonia models' confusion matrices based on the training using 70% of the pneumonia slices. (b) The pneumonia models' confusion matrices based on the training using 50% of the pneumonia slices. (c) The pneumonia models' confusion matrices based on the training using 30% of the pneumonia slices.

**Figure 13:**
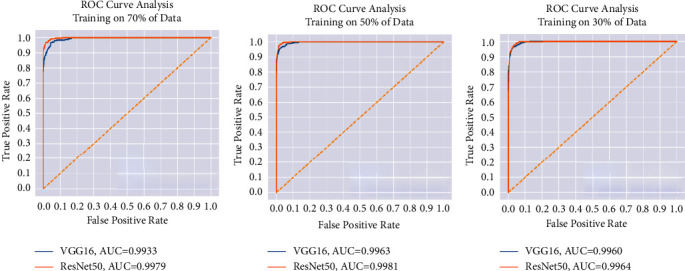
The pneumonia models' ROC for each training scenario. (a) ROC when training using 70% of the pneumonia slices. (b) ROC when training using 50% of the pneumonia slices. (c) ROC when training using 30% of the pneumonia slices.

**Figure 14:**
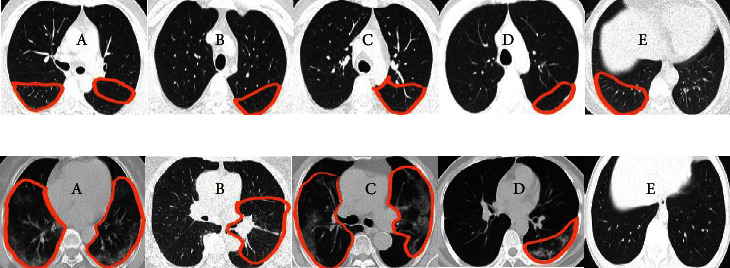
Examples of misclassified slices by pneumonia DSVDD-VGG and DSVDD-ResNet models. All areas marked represent small area of GGO. (a). Pneumonia slices were classified as non-pneumonia. (b). Non-pneumonia slices were classified as pneumonia. Marked areas represent in (A) GGO (B) FB (C, D) GGO (E) healthy slice.

Table 5 summarizes the pneumonia models' validation and testing evaluation metrics: specificity, sensitivity, F1 score, and AUC. As shown in the confusion matrices in Figure 12, DSVDD-ResNet outperformed DSVDD-VGG, such that DSVDD-ResNet was very sensitive to pneumonia, with only eight slices misclassified using the model trained on 70% of the data and only 20 slices misclassified using the model trained on 30% of the data. Furthermore, DSVDD-VGG correctly classified the majority of pneumonia CTs, with only (8, 21, and 28) misclassified among the three DSVDD-VGG models.  Figure 13 depicts the pneumonia models' ROC curves, which show the models' stability and ability to achieve high performance in the absence of a large dataset.  Figure 14 depicts examples of misclassified slices.

We provided a quantitative comparison of the average metrics values for DSVDD-VGG and DSVDD-ResNet models based on all the training scenarios with the state-of-the-art methods in Table 6.

As shown in Table 6, DSVDD-ResNet50 outperformed the majority of the state-of-the-art methods used in the comparison and achieved the highest metric values among the majority of metrics used. We excluded from the comparison studies that used only two classes or that used tumor data, where distinguishing between tumor and COVID-19 is simple due to the distinct signs of tumor.

#### 6.3.4. Statistical Analysis Using McNemar's Test

We devoted this section to comparing the performance of the proposed models using McNemar's test. McNemar is an Statistical hypothesis test that can be used to evaluate two binary classifiers. It performs the test using a 2 × 2 contingency table [47]. We have two hypotheses we are going to investigate using this test:

*H *_0_ = The Null Hypothesis: both classifiers have the same error rate.

*H *_1_ = The alternative hypothesis, a significant difference exists and both have a different error rate.

McNemar statistical values are computed as follows:

(7) $$\begin{array}{c} Mcnemar\;statistics\;x^{2}=\frac{{\left(n_{10}-n_{01}\right)}^{2}}{\left(n_{10}+n_{01}\right)}\text{.} \end{array}$$

Such that:

*n*_10_ = The number of correctly classified samples by ResNet50 but misclassified by VGG16.

*n*_01_ = The number of correctly classified samples by VGG16 but misclassified by ResNet50

Tables 7–9 contain the 2 × 2 contingency tables for all models' comparisons and the results of performing the McNemar test with significance threshold *α*=0.05.

### 6.4. Discussion

Although COVID-19 is a new strain of coronavirus and there is not enough data, many studies have proposed many deep models to diagnose the disease and aid in the fight against it. In our turn, we proposed a new approach to discriminate between COVID-19, non-COVID pneumonia, and intact lung CTs. The proposed framework is different from the other studies in that a one-class deep model is devoted to diagnosing each class in our dataset, which overcome the imbalance-class problem.

**Figure 15:**
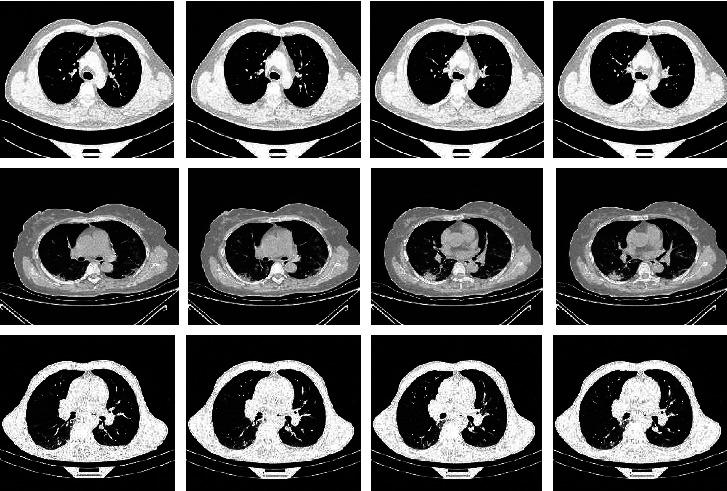
From top to bottom, three rows of consecutive slices for intact, COVID-19, and non-COVID pneumonia CTs. The first and second slices in each row are very similar.

Although the results of the proposed framework were promising, we noticed some important issues that needed to be discussed. In the COVID-19 models, the trained models using 30% of the data produced the same AUC values as the models trained on 70% of the data and were better than the models trained on 50%. This is because the CT scan files are composed of a set of slices, and consecutive slices are very similar (see Figure 15). To overcome this problem, we should use nonconsecutive slices or use slices from different CT files for different patients during the construction of the dataset, but in our situation, we did not have enough data.

**Figure 16:**
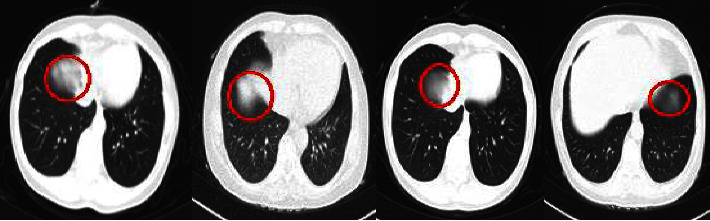
The red-circled areas in these intact slices are similar to the consolidation areas in the pneumonia CTs.

In addition, the intact model's performance was lower than the COVID-19 and pneumonia models due to some area around the parenchyma are similar to the GGO, which is the main indicator of pneumonia (see Figure 16).

**Figure 17:**
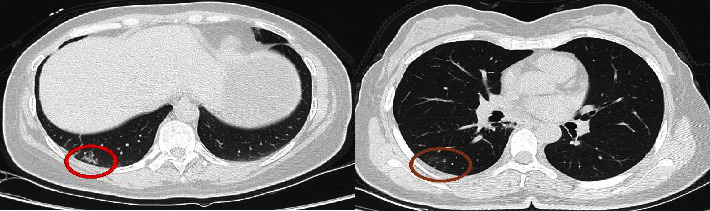
Because the lesions were on the edges (red-circled areas), COVID-19 was not detected in these slices.

Moreover, the proposed models sometimes failed to identify the infected people when the lesion was at the edge of the lung's parenchyma, especially in the slices at the beginning or end of the CT files (see Figure 17). Therefore, we suggest considering all the slices of each patient as one instance in order to solve this issue.

## 7. Conclusion

This study proposed a new approach for distinguishing COVID-19 from other infections in an effort to halt the disease's spread. Several models are included in the proposed approach, including one that classifies intact lung images as normal and the others as anomalous; another that identifies the COVID-19-infected lung and the other images as anomalous; and finally, a model that identifies the lung with non-COIVD-19 pneumonia and the other images as anomalous. Our approach models were built using DSVDD and the pretrained models VGG16 and ResNet50. The VGG16 and ResNet50 models are used separately to be the deep network of DSVDD, such that for every category in our dataset, two models are built: one combining DSVDD with ResNet50 and the other combining VGG16 with DSVDD. We trained all models in an end-to-end fashion and used different split ratios of data for more verification of the efficiency of our approach. Experiment results revealed that the majority of proposed models outperformed state-of-the-art models. In future work, we will try to combine the patient's symptoms, if they are available, with the extracted features to improve the outcome. Furthermore, we will employ semantic segmentation to detect lesion areas and assess the severity of the infection.

## Figures and Tables

**Table 1:** A summary of the related work, including the methodology, dataset, and evaluation metrics.

Study	Number of samples	Methodology	Performance criteria
[19]	COVID-19 = 368	Deep fusion model based on Resnet50 and multiview CT image inputs	AUC = 0.819
	Non-COVID = 127		Accuracy = 0.76
			Sensitivity = 0.73

[20]	COVID-19 = 510	Ten pretrained CNN models were used to classify CT images into COVID and non-COVID	AUC = 0.994–0.999
			Sensitivity = 100
	Non-COVID = 510		Specificity = 99.02–99.26
			Accuracy = 99.51–99.63

[21]	COVID-19 = 449	Three-task model for classification, lesion segmentation, and reconstruction tasks	Sensitivity = 96
	Non-COVID = 495		Specificity = 92
	Normal = 425		Accuracy = 94.67

[22]	COVID-19 = 829	Pretrained ResNet50 is used to classify the ROIs as normal or abnormal	AUC = 0.994
			Sensitivity = 0.94
	Normal = 1036		Specificity = 0.98

[23]	COVID-19 = 64,771	Model based on Res2Net	Sensitivity = 0.95
	Non-COVID = 75,541		Specificity = 0.93

[24]	COVID-19 = 706	Model based on pretrained EfficientNetB3	Sensitivity = 0.924
	Non-COVID = 764		Specificity = 0.983
	Normal = 654		Accuracy = 0.964
			F1 score = 0.953 (COVID vs other classes)

[25]	COVID-19 = 1315	A model consists of prior attention blocks is proposed. The attention block consists of two branches: one that detects pneumonia and another that determines the type of pneumonia found	Sensitivity = 0.876
	Non-COVID = 2406		Specificity = 0.955
	Normal = 936		Accuracy = 0.932

[26]	450 CT files for COVID-19, non-COVID, and normal patients	A model inspired by VGG and based on weakly supervised learning is proposed. To improve accuracy, the model concatenated multiple scaled features from various levels	AUC = 0.895
			Sensitivity = 88.5
			Specificity = 87.1
			Accuracy = 0.874

[27]	COVID-19 = 1296	3D deep model based on ResNet50	AUC = 0.963
	Non-COVID = 1753		Sensitivity = 0.903
	Normal = 1325		Specificity = 0.946

[28]	COVID-19 = 325	A modified inception transfer-learning model	Sensitivity = 0.67
	Non-COVID = 740		Specificity = 0.83
			Accuracy = 0.793

[29]	COVID-19 = 2500	An ensemble model combining GoogleNet, ResNet, and AlexNet pretrained models. The relative major vote is used to determine the classification result	Sensitivity = 0.9905
	Tumor = 2500		Specificity = 0.996
	Normal = 2500		F score = 0.9859

[30]	COVID-19 = 94548	A model based on VGG19 and transformer	Precision = 0.9745
	Non-COVID = 40291		Recall = 0.9776
			Specificity = 0.9601
	Normal = 60053		Accuracy = 0.9673
			F score = 0.9636

**Table 2:** The details about the employed datasets.

Dataset	Intact	Pneumonia	COVID-19	Non-COVID illness
COVID_DATASET	2495	1825	719	—
SARS-CoV-2	—	—	1252	1230
COVID-CT	—	—	349	397
Ours	1556	1203	1944	—

**Table 3:** The intact DSVDD-VGG and DSVDD-ResNet models' evaluation metrics.

Samples ratio (%)	Network model	Validation				Testing			
		Specificity	Sensitivity	F1 scores	AUC	Specificity	Sensitivity	F1 scores	AUC
70	VGG	0.9713	0.9290	0.9351	0.9873	0.9412	**0.9840**	0.7637	**0.9947**
	ResNet	**0.9904**	0.9097	**0.9431**	0.9863	**0.9790**	0.9551	0.8817	0.9938

50	VGG	0.9554	0.9484	0.9304	**0.9903**	0.9422	0.9647	0.8549	0.9889
	ResNet	0.9713	**0.9613**	0.9521	0.9894	0.9520	0.9454	0.8643	0.9870

30	VGG	0.9395	0.9548	0.9193	0.9881	0.9431	0.9273	0.8753	0.9837
	ResNet	0.9427	0.9548	0.9221	0.9899	0.9701	0.8984	**0.8989**	0.9790

The bold value represents the highest value in each column.

**Table 4:** The COVID-19 DSVDD-VGG and DSVDD-ResNet models' evaluation metrics.

Samples ratio (%)	Network model	Validation				Testing			
		Specificity	Sensitivity	F1 scores	AUC	Specificity	Sensitivity	F1 scores	AUC
70	VGG	**1.0**	0.9897	0.9948	0.9999	0.9957	0.9897	0.9797	**0.9996**
	ResNet	0.9927	**0.9948**	0.9923	0.9994	0.9906	0.9949	0.9652	**0.9996**

50	VGG	**1.0**	**0.9948**	0.9974	0.9999	**0.9978**	0.9923	0.9923	0.9993
	ResNet	0.9964	0.9897	0.9922	0.9997	0.9841	**0.9974**	0.9712	0.9994

30	VGG	0.9927	**0.9948**	0.9923	0.9999	0.9942	**0.9974**	0.9919	**0.9996**
	ResNet	0.9964	0.9897	0.9922	0.9992	0.9877	0.9914	**0.9813**	0.9991

The bold value represents the highest value in each column.

**Table 5:** The pneumonia DSVDD-VGG and DSVDD-ResNet models' evaluation metrics.

Samples ratio (%)	Network model	Validation				Testing			
		Specificity	Sensitivity	F1 scores	AUC	Specificity	Sensitivity	F1 scores	AUC
70	VGG	0.9685	0.9500	0.9306	0.9951	0.9557	0.9668	0.7409	0.9933
	ResNet	0.9828	0.9833	0.9672	0.9987	**0.9851**	0.9668	0.8859	0.9979

50	VGG	0.9828	0.9667	0.9587	0.9978	0.9780	0.9564	0.9039	0.9963
	ResNet	**0.9857**	**0.9917**	**0.9754**	**0.9987**	0.9797	**0.9793**	0.9210	**0.9981**

30	VGG	0.9799	0.9583	0.9504	0.9962	0.9740	0.9613	**0.9211**	0.9960
	ResNet	0.9542	0.9750	0.9249	0.9943	0.9706	0.9723	0.9196	0.9964

**Table 6:** A quantitative comparison of the state-of-the-art methods with the all-proposed models.

Method	Specificity	Sensitivity	F1 scores	AUC	Accuracy
Yousefzadeh et al. [24]	0.983	0.924	0.953	—	0.964
Wang et al. [25]	0.955	0.876	—	—	0.932
Hu et al. [26]	87.1	88.5	—	0.895	0.87.4
Li [27]	0.946	0.903	—	0.963	—
Wang et al. [28]	0.83	0.67	—	—	0.793
Fan et al. [30]	0.9601	0.9776	**0.9636**	—	0.9673
DSVDD-VGG16 70% training samples	0.9642	**0.9802**	0.8281	0.9958	—
DSVDD-VGG16 50% training samples	0.9727	0.9711	0.9170	0.9948	—
DSVDD-VGG16 30% training samples	0.9704	0.962	0.9294	0.9931	—
DSVDD-ResNet50 70% training samples	**0.9849**	0.9723	0.9109	**0.9971**	—
DSVDD- ResNet50 50% training samples	0.9719	0.9740	0.9188	0.9948	—
DSVDD- ResNet50 30% training samples	0.9761	0.9540	0.9333	0.9915	—

The bold value represents the highest value in each column.

**Table 7:** The contingency tables of the normal models and McNemar test results according to all partitions used.

	VGG16								
	Training on 70%			Training on 50%			Training on 30%		
ReSNet50		0	1		0	1		0	1
	0	51	29	0	105	80	0	90	99
	1	139	3240	1	99	3486	1	157	3736

McNemar	72.023810			2.016760			13.140625		
*p* value	0.000000 < 0.05			0.155571 > 0.05			0.000289 < 0.05		
Rejecting the null hypothesis				Accepting the null hypothesis			Rejecting the null hypothesis		

**Table 8:** The contingency tables of the COVID-19 models and McNemar test results according to all partitions used.

	VGG16								
	Training on 70%			Training on 50%			Training on 30%		
ReSNet50		0	1		0	1		0	1
	0	5	23	0	3	43	0	7	37
	1	11	3110	1	9	3482	1	12	3870

McNemar	4.235			22.23			12.755		
*p* value	0.039592 < 0.05			0.000003 < 0.05			0.000355 < 0.05		
Rejecting the null hypothesis				Rejecting the null hypothesis			Rejecting the null hypothesis		

**Table 9:** The contingency tables of the pneumonia models and McNemar test results according to all partitions used.

	VGG16								
	Training on 70%			Training on 50%			Training on 30%		
		0	1		0	1		0	1
ReSNet50	0	26	34	0	33	48	0	57	66
	1	137	3544	1	65	3836	1	62	4038

McNemar	62.04			2.5575			0.125000		
*p* value	0.000000 < 0.05			0.10977051 > 0.05			0.723674 > 0.05		
Rejecting the null hypothesis				Accepting the null hypothesis			Accepting the null hypothesis		

## Data Availability

The data used in this study are provided in the reference.

## References

[1] Organization W. H. (2019). Coronavirus disease (COVID-19). https://www.who.int/health-topics/coronavirus#tab=tab_1.

[2] Zhu N., Zhang D., Wang W. (2019). A novel coronavirus from patients with pneumonia in China. *New England Journal of Medicine*.

[3] Huang C., Wang Y., Li X. (2020). Clinical features of patients infected with 2019 novel coronavirus in Wuhan, China. *Lancet*.

[4] Lotfy M., El-Bakry H., Elgayar M. (2022). Semantic pneumonia segmentation and classification for covid-19 using deep learning network. *Computers, Materials and Continua*.

[5] Hui D. S., I Azhar E., Madani T. A. (2020). The continuing 2019-nCoV epidemic threat of novel coronaviruses to global health—the latest 2019 novel coronavirus outbreak in Wuhan, China. *International Journal of Infectious Diseases*.

[6] Shahi T., Sitaula C., Paudel N. (2022). A hybrid feature extraction method for Nepali COVID-19-related tweets classification. *Computational Intelligence and Neuroscience*.

[7] Sitaula C., Basnet A., Mainali A., Shahi T. B. (2021). Deep learning-based methods for sentiment analysis on Nepali covid-19-related tweets. *Computational Intelligence and Neuroscience*.

[8] Sitaula C., Shahi T. B. (2022). Multi-channel CNN to classify Nepali covid-19 related tweets using hybrid features. https://arxiv.org/abs/2203.10286.

[9] Bhandari M., Shahi T. B., Siku B., Neupane A. (2022). Explanatory classification of CXR images into COVID-19, Pneumonia and Tuberculosis using deep learning and XAI. *Computers in Biology and Medicine*.

[10] Long C., Xu H., Shen Q. (2020). Diagnosis of the Coronavirus disease (COVID-19): rRT-PCR or CT?. *European Journal of Radiology*.

[11] Khan A., Khan S. H., Saif M., Batool A., Sohail A., Khan M. W. (2022). A survey of deep learning techniques for the analysis of COVID-19 and their usability for detecting omicron. https://arxiv.org/abs/2202.06372.

[12] Rahimzadeh M., Attar A., Sakhaei S. M. (2021). A fully automated deep learning-based network for detecting covid-19 from a new and large lung ct scan dataset. *Biomedical Signal Processing and Control*.

[13] Litjens G., Kooi T., Bejnordi B. E. (2017). A survey on deep learning in medical image analysis. *Medical Image Analysis*.

[14] El-Rashidy N., El-Sappagh S., Islam S. M. R., El-Bakry H. M., Abdelrazek S. (2020). End-to-end deep learning framework for coronavirus (COVID-19) detection and monitoring. *Electronics*.

[15] Javaheri T. (2020). Covidctnet: an open-source deep learning approach to identify covid-19 using ct image. https://arxiv.org/abs/2005.03059.

[16] Khan A. I., Shah J. L., Bhat M. M. (2020). CoroNet: a deep neural network for detection and diagnosis of COVID-19 from chest x-ray images. *Computer Methods and Programs in Biomedicine*.

[17] Subramanian N., Elharrouss O., Al-Maadeed S., Chowdhury M. (2022). A review of deep learning-based detection methods for COVID-19. *Computers in Biology and Medicine*.

[18] Shen D., Wu G., Suk H.-I. (2017). Deep learning in medical image analysis. *Annual Review of Biomedical Engineering*.

[19] Wu X., Hui H., Niu M. (2020). Deep learning-based multi-view fusion model for screening 2019 novel coronavirus pneumonia: a multicentre study. *European Journal of Radiology*.

[20] Ardakani A. A., Kanafi A. R., Acharya U. R., Khadem N., Mohammadi A. (2020). Application of deep learning technique to manage COVID-19 in routine clinical practice using CT images: results of 10 convolutional neural networks. *Computers in Biology and Medicine*.

[21] Amyar A., Modzelewski R., Li H., Ruan S. (2020). Multi-task deep learning based CT imaging analysis for COVID-19 pneumonia: classification and segmentation. *Computers in Biology and Medicine*.

[22] Gozes O., Frid-Adar M., Sagie N., Zhang H., Ji W., Greenspan H. (2020). Coronavirus detection and analysis on chest ct with deep learning. https://arxiv.org/abs/2004.02640.

[23] Wu Y.-H., Gao S. H., Mei J. (2021). Jcs: an explainable covid-19 diagnosis system by joint classification and segmentation. *IEEE Transactions on Image Processing*.

[24] Yousefzadeh M., Esfahanian P., Movahed S. M. S. (2021). Correction: ai-corona: radiologist-assistant deep learning framework for COVID-19 diagnosis in chest CT scans. *PLoS One*.

[25] Wang J., Bao Y., Wen Y. (2020). Prior-attention residual learning for more discriminative COVID-19 screening in CT images. *IEEE Transactions on Medical Imaging*.

[26] Hu S., Gao Y., Niu Z. (2020). Weakly supervised deep learning for covid-19 infection detection and classification from ct images. *IEEE Access*.

[27] Li L., Qin L., Xu Z. (2020). Using artificial intelligence to detect COVID-19 and community-acquired pneumonia based on pulmonary CT: evaluation of the diagnostic accuracy. *Radiology*.

[28] Wang S., Kang B., Ma J. (2021). A deep learning algorithm using CT images to screen for Corona Virus Disease (COVID-19). *European Radiology*.

[29] Zhou T., Lu H., Yang Z., Qiu S., Huo B., Dong Y. (2021). The ensemble deep learning model for novel COVID-19 on CT images. *Applied Soft Computing*.

[30] Fan X., Feng X., Dong Y., Hou H. (2022). COVID-19 CT image recognition algorithm based on transformer and CNN. *Displays*.

[31] Ruff L. Deep one-class classification.

[32] Bhardwaj A., Di W., Wei J. (2018). *Deep Learning Essentials: Your Hands-On Guide to the Fundamentals of Deep Learning and Neural Network Modeling*.

[33] Glassner A. (2018). Deep learning: from basics to practice. *The Imaginary Institute*.

[34] Apostolopoulos I. D., Mpesiana T. A. (2020). Covid-19: automatic detection from x-ray images utilizing transfer learning with convolutional neural networks. *Physical and engineering sciences in medicine*.

[35] Huh M., Agrawal P., Efros A. A. (2016). What makes ImageNet good for transfer learning?. https://arxiv.org/abs/1608.08614.

[36] Han Z., Wei B., Hong Y. (2020). Accurate screening of COVID-19 using attention-based deep 3D multiple instance learning. *IEEE Transactions on Medical Imaging*.

[37] Zhao J. a. Z., Yichen, He X., Xie P. (2020). COVID-CT-Dataset: a CT scan dataset about COVID-19. https://github.com/UCSD-AI4H/COVID-CT/tree/master/Images-processed.

[38] Zeynaloy (2020). Covid19 CT images dataset. https://www.kaggle.com/zeynaloy/covid19-ct-images-dataset.

[39] Eduardo P. (2020). SARS-COV-2 Ct-Scan dataset. https://www.kaggle.com/plameneduardo/sarscov2-ctscan-dataset.

[40] Ma J. (2020). COVID-19 CT lung and infection segmentation dataset. https://zenodo.org/record/3757476#.Yab9b9BBxPa.

[41] Ronneberger O., Fischer P., Brox T. U-net: convolutional networks for biomedical image segmentation.

[42] He K., Zhang X., Ren S., Sun J. Deep residual learning for image recognition.

[43] Simonyan K., Zisserman A. (2014). Very deep convolutional networks for large-scale image recognition. https://arxiv.org/abs/1409.1556.

[44] Sitaula C., Shahi T. B., Aryal S., Marzbanrad F. (2021). Fusion of multi-scale bag of deep visual words features of chest X-ray images to detect COVID-19 infection. *Scientific Reports*.

[45] Sitaula C., Shahi T. B. (2022). Monkeypox virus detection using pre-trained deep learning-based approaches. *Journal of Medical Systems*.

[46] Alshazly H., Linse C., Barth E., Martinetz T. (2021). Explainable COVID-19 detection using chest CT scans and deep learning. *Sensors*.

[47] Wikipedia (2022). McNemar’s test. https://en.wikipedia.org/wiki/McNemar%27s_test.

